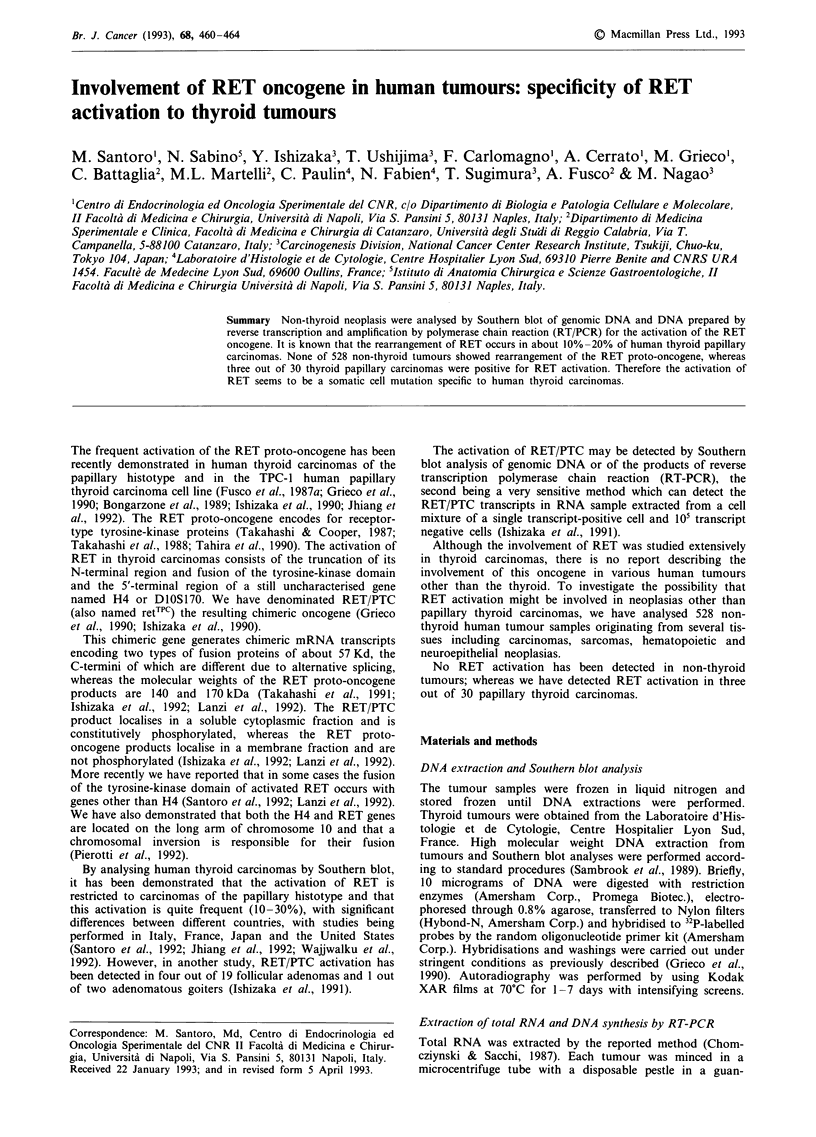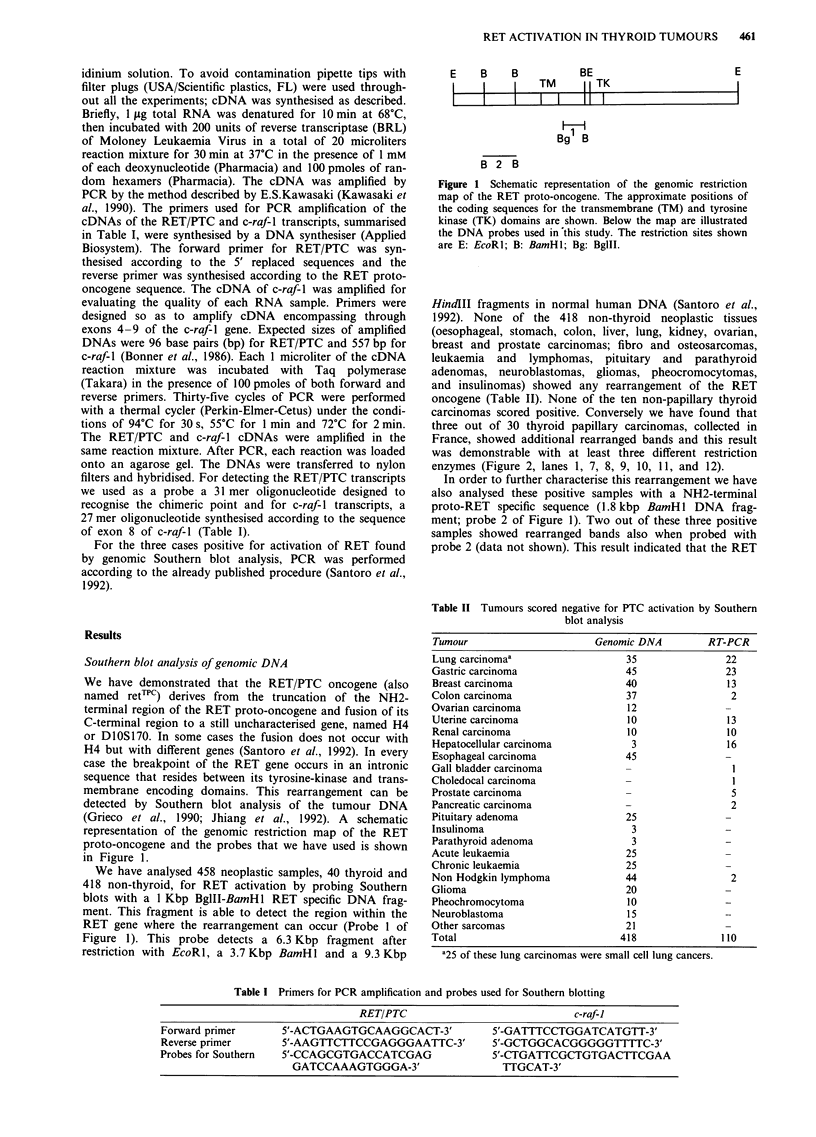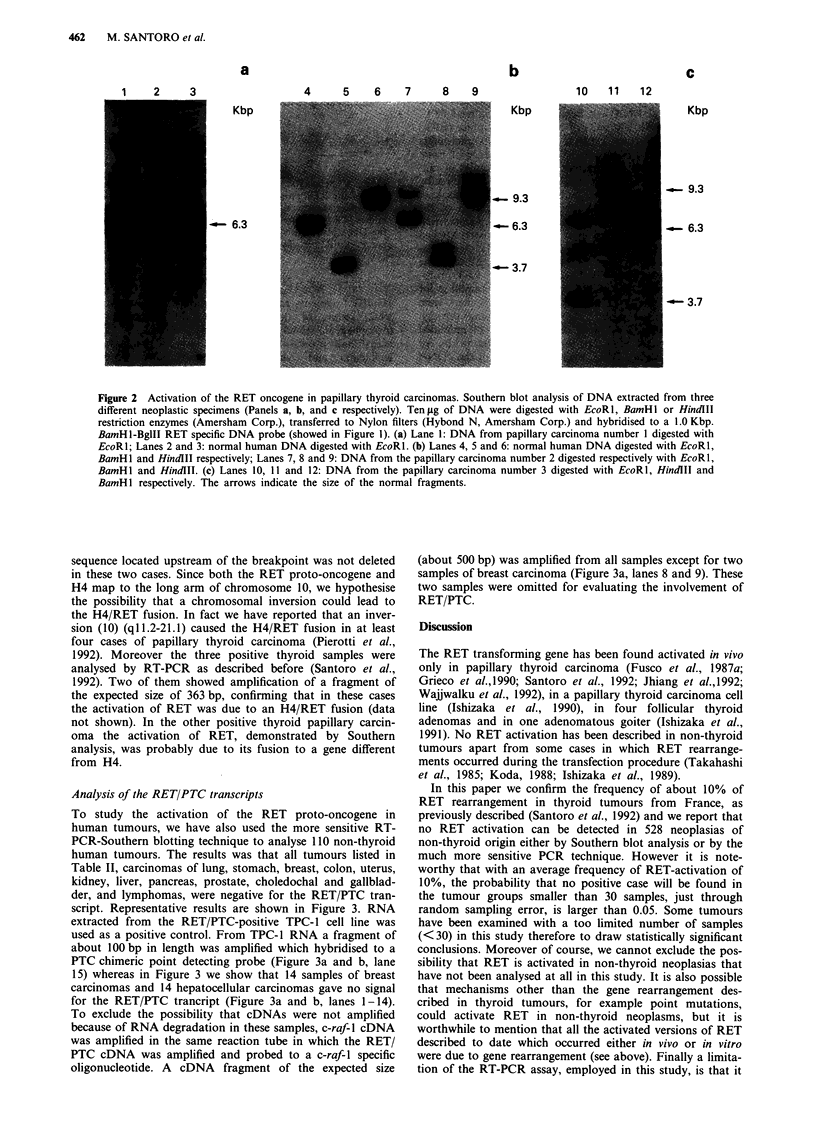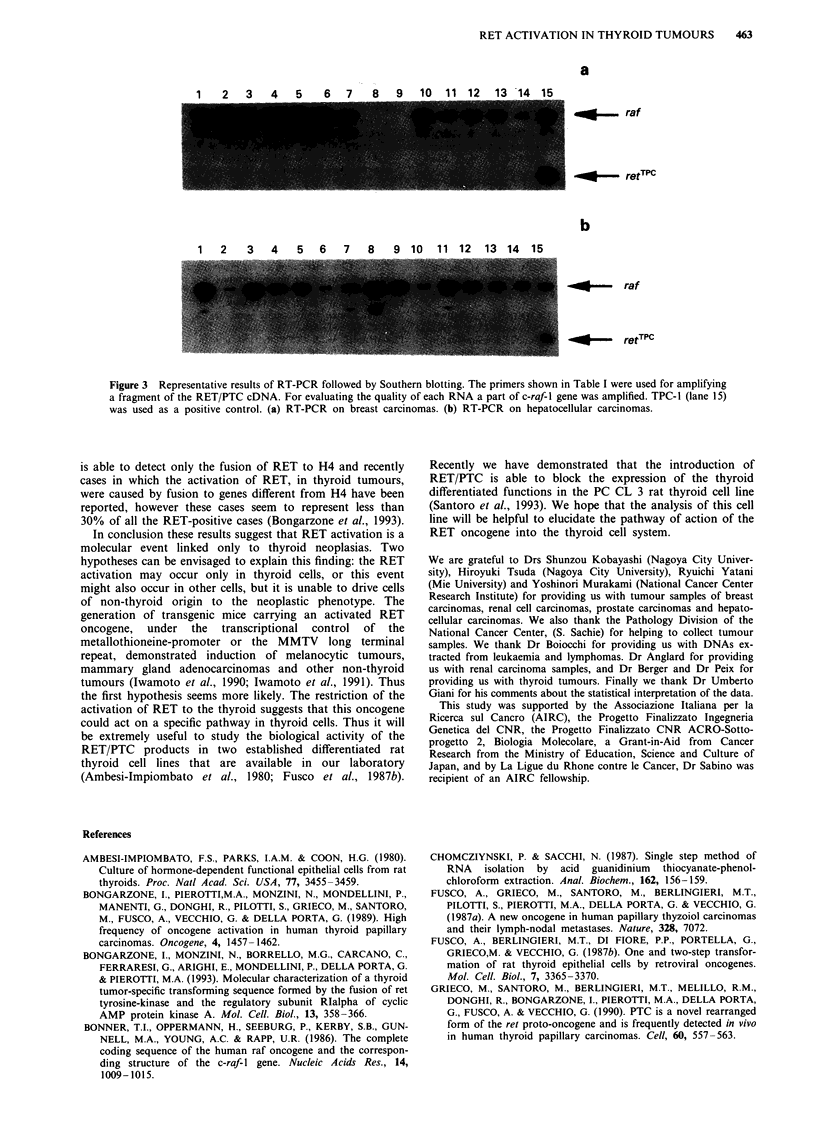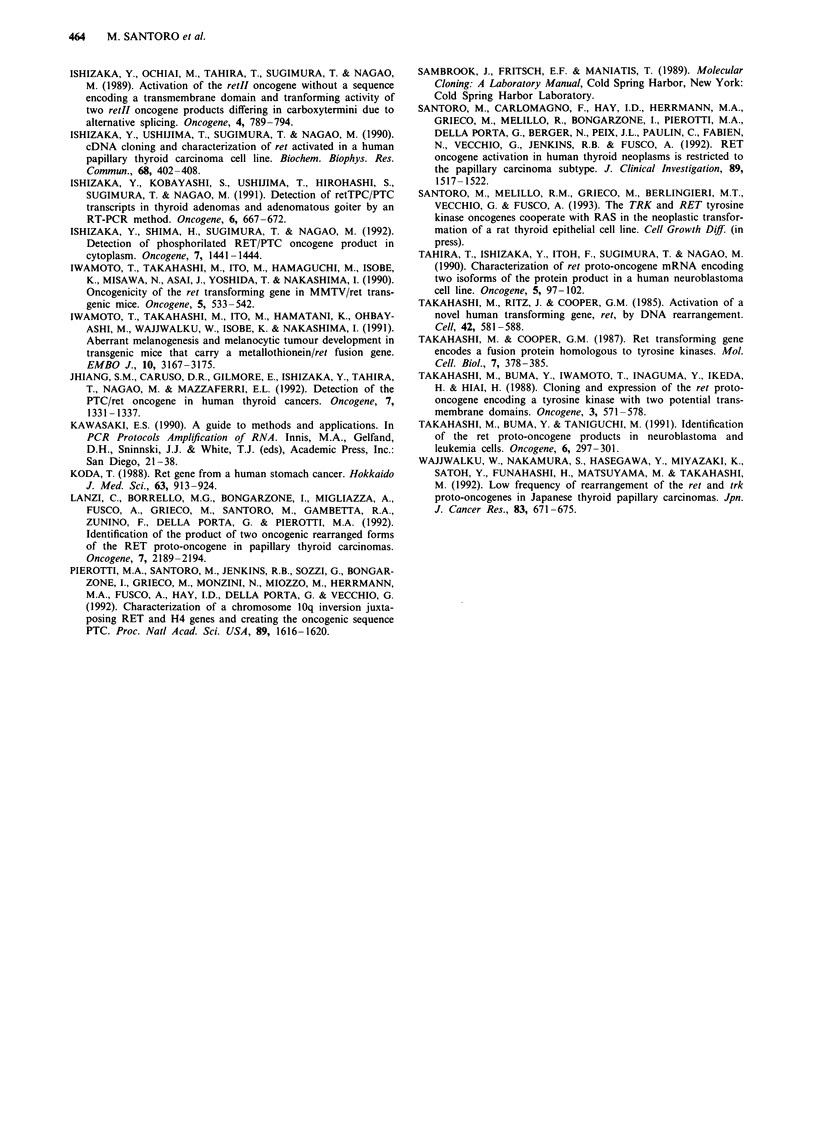# Involvement of RET oncogene in human tumours: specificity of RET activation to thyroid tumours.

**DOI:** 10.1038/bjc.1993.370

**Published:** 1993-09

**Authors:** M. Santoro, N. Sabino, Y. Ishizaka, T. Ushijima, F. Carlomagno, A. Cerrato, M. Grieco, C. Battaglia, M. L. Martelli, C. Paulin

**Affiliations:** Centro di Endocrinologia ed Oncologia Sperimentale del CNR, Dipartimento di Biologia e Patologia Cellulare e Molecolare, II Facoltà di Medicina e Chirurgia, Università di Napoli, Italy.

## Abstract

**Images:**


Br. J. Cancer (1993), 68, 460–464 © Macmillan Press Ltd., 1993

### Involvement of RET oncogene in human tumours: specificity of RET activation to thyroid tumours

M. Santoro[1], N. Sabino[5], Y. Ishizaka[3], T. Ushijima[3], F. Carlomagno[1], A. Cerrato[1], M. Grieco[1], C. Battaglia[2], M.L. Martelli[2], C. Paulin[4], N. Fabien[4], T. Sugimura[3], A. Fusco[2] & M. Nagao[3]

[1]*Centro di Endocrinologia ed Oncologia Sperimentale del CNR, c/o Dipartimento di Biologia e Patologia Cellulare e Molecolare, II Facoltà di Medicina e Chirurgia, Università di Napoli, Via S. Pansini 5, 80131 Naples, Italy;* [2]*Dipartimento di Medicina Sperimentale e Clinica, Facoltà di Medicina e Chirurgia di Catanzaro, Università degli Studi di Reggio Calabria, Via T. Campanella, 5-88100 Catanzaro, Italy;* [3]*Carcinogenesis Division, National Cancer Center Research Institute, Tsukiji, Chuo-ku, Tokyo 104, Japan;* [4]*Laboratoire d'Histologie et de Cytologie, Centre Hospitalier Lyon Sud, 69310 Pierre Benite and CNRS URA 1454. Faculté de Medecine Lyon Sud, 69600 Oullins, France;* [5]*Istituto di Anatomia Chirurgica e Scienze Gastroentologiche, II Facoltà di Medicina e Chirurgia Università di Napoli, Via S. Pansini 5, 80131 Naples, Italy.*

**Summary** Non-thyroid neoplasis were analysed by Southern blot of genomic DNA and DNA prepared by reverse transcription and amplification by polymerase chain reaction (RT/PCR) for the activation of the RET oncogene. It is known that the rearrangement of RET occurs in about 10%–20% of human thyroid papillary carcinomas. None of 528 non-thyroid tumours showed rearrangement of the RET proto-oncogene, whereas three out of 30 thyroid papillary carcinomas were positive for RET activation. Therefore the activation of RET seems to be a somatic cell mutation specific to human thyroid carcinomas.

The frequent activation of the RET proto-oncogene has been recently demonstrated in human thyroid carcinomas of the papillary histotype and in the TPC-1 human papillary thyroid carcinoma cell line (Fusco *et al.*, 1987*a*; Grieco *et al.*, 1990; Bongarzone *et al.*, 1989; Ishizaka *et al.*, 1990; Jhiang *et al.*, 1992). The RET proto-oncogene encodes for receptor-type tyrosine-kinase proteins (Takahashi & Cooper, 1987; Takahashi *et al.*, 1988; Tahira *et al.*, 1990). The activation of RET in thyroid carcinomas consists of the truncation of its N-terminal region and fusion of the tyrosine-kinase domain and the 5′-terminal region of a still uncharacterised gene named H4 or D10S170. We have denominated RET/PTC (also named $ret^{TPC}$) the resulting chimeric oncogene (Grieco *et al.*, 1990; Ishizaka *et al.*, 1990).

This chimeric gene generates chimeric mRNA transcripts encoding two types of fusion proteins of about 57 Kd, the C-termini of which are different due to alternative splicing, whereas the molecular weights of the RET proto-oncogene products are 140 and 170 kDa (Takahashi *et al.*, 1991; Ishizaka *et al.*, 1992; Lanzi *et al.*, 1992). The RET/PTC product localises in a soluble cytoplasmic fraction and is constitutively phosphorylated, whereas the RET proto-oncogene products localise in a membrane fraction and are not phosphorylated (Ishizaka *et al.*, 1992; Lanzi *et al.*, 1992). More recently we have reported that in some cases the fusion of the tyrosine-kinase domain of activated RET occurs with genes other than H4 (Santoro *et al.*, 1992; Lanzi *et al.*, 1992). We have also demonstrated that both the H4 and RET genes are located on the long arm of chromosome 10 and that a chromosomal inversion is responsible for their fusion (Pierotti *et al.*, 1992).

By analysing human thyroid carcinomas by Southern blot, it has been demonstrated that the activation of RET is restricted to carcinomas of the papillary histotype and that this activation is quite frequent (10–30%), with significant differences between different countries, with studies being performed in Italy, France, Japan and the United States (Santoro *et al.*, 1992; Jhiang *et al.*, 1992; Wajjwalku *et al.*, 1992). However, in another study, RET/PTC activation has been detected in four out of 19 follicular adenomas and 1 out of two adenomatous goiters (Ishizaka *et al.*, 1991).

Correspondence: M. Santoro, Md, Centro di Endocrinologia ed Oncologia Sperimentale del CNR II Facoltà di Medicina e Chirurgia, Università di Napoli, Via S. Pansini 5, 80131 Napoli, Italy.
Received 22 January 1993; and in revised form 5 April 1993.

The activation of RET/PTC may be detected by Southern blot analysis of genomic DNA or of the products of reverse transcription polymerase chain reaction (RT-PCR), the second being a very sensitive method which can detect the RET/PTC transcripts in RNA sample extracted from a cell mixture of a single transcript-positive cell and $10^5$ transcript negative cells (Ishizaka *et al.*, 1991).

Although the involvement of RET was studied extensively in thyroid carcinomas, there is no report describing the involvement of this oncogene in various human tumours other than the thyroid. To investigate the possibility that RET activation might be involved in neoplasias other than papillary thyroid carcinomas, we have analysed 528 non-thyroid human tumour samples originating from several tissues including carcinomas, sarcomas, hematopoietic and neuroepithelial neoplasias.

No RET activation has been detected in non-thyroid tumours; whereas we have detected RET activation in three out of 30 papillary thyroid carcinomas.

### Materials and methods

### *DNA extraction and Southern blot analysis*

The tumour samples were frozen in liquid nitrogen and stored frozen until DNA extractions were performed. Thyroid tumours were obtained from the Laboratoire d'Histologie et de Cytologie, Centre Hospitalier Lyon Sud, France. High molecular weight DNA extraction from tumours and Southern blot analyses were performed according to standard procedures (Sambrook *et al.*, 1989). Briefly, 10 micrograms of DNA were digested with restriction enzymes (Amersham Corp., Promega Biotec.), electrophoresed through 0.8% agarose, transferred to Nylon filters (Hybond-N, Amersham Corp.) and hybridised to $^{32}P$-labelled probes by the random oligonucleotide primer kit (Amersham Corp.). Hybridisations and washings were carried out under stringent conditions as previously described (Grieco *et al.*, 1990). Autoradiography was performed by using Kodak XAR films at 70°C for 1–7 days with intensifying screens.

### *Extraction of total RNA and DNA synthesis by RT-PCR*

Total RNA was extracted by the reported method (Chomczyinski & Sacchi, 1987). Each tumour was minced in a microcentrifuge tube with a disposable pestle in a guanidinium solution. To avoid contamination pipette tips with filter plugs (USA/Scientific plastics, FL) were used throughout all the experiments; cDNA was synthesised as described. Briefly, 1 μg total RNA was denatured for 10 min at 68°C, then incubated with 200 units of reverse transcriptase (BRL) of Moloney Leukaemia Virus in a total of 20 microliters reaction mixture for 30 min at 37°C in the presence of 1 mM of each deoxynucleotide (Pharmacia) and 100 pmoles of random hexamers (Pharmacia). The cDNA was amplified by PCR by the method described by E.S.Kawasaki (Kawasaki *et al.*, 1990). The primers used for PCR amplification of the cDNAs of the RET/PTC and c-*raf*-1 transcripts, summarised in Table I, were synthesised by a DNA synthesiser (Applied Biosystem). The forward primer for RET/PTC was synthesised according to the 5′ replaced sequences and the reverse primer was synthesised according to the RET proto-oncogene sequence. The cDNA of c-*raf*-1 was amplified for evaluating the quality of each RNA sample. Primers were designed so as to amplify cDNA encompassing through exons 4–9 of the c-*raf*-1 gene. Expected sizes of amplified DNAs were 96 base pairs (bp) for RET/PTC and 557 bp for c-*raf*-1 (Bonner *et al.*, 1986). Each 1 microliter of the cDNA reaction mixture was incubated with Taq polymerase (Takara) in the presence of 100 pmoles of both forward and reverse primers. Thirty-five cycles of PCR were performed with a thermal cycler (Perkin-Elmer-Cetus) under the conditions of 94°C for 30 s, 55°C for 1 min and 72°C for 2 min. The RET/PTC and c-*raf*-1 cDNAs were amplified in the same reaction mixture. After PCR, each reaction was loaded onto an agarose gel. The DNAs were transferred to nylon filters and hybridised. For detecting the RET/PTC transcripts we used as a probe a 31 mer oligonucleotide designed to recognise the chimeric point and for c-*raf*-1 transcripts, a 27 mer oligonucleotide synthesised according to the sequence of exon 8 of c-*raf*-1 (Table I).

For the three cases positive for activation of RET found by genomic Southern blot analysis, PCR was performed according to the already published procedure (Santoro *et al.*, 1992).

### Results

### *Southern blot analysis of genomic DNA*

We have demonstrated that the RET/PTC oncogene (also named $ret^{TPC}$) derives from the truncation of the NH2-terminal region of the RET proto-oncogene and fusion of its C-terminal region to a still uncharacterised gene, named H4 or D10S170. In some cases the fusion does not occur with H4 but with different genes (Santoro *et al.*, 1992). In every case the breakpoint of the RET gene occurs in an intronic sequence that resides between its tyrosine-kinase and transmembrane encoding domains. This rearrangement can be detected by Southern blot analysis of the tumour DNA (Grieco *et al.*, 1990; Jhiang *et al.*, 1992). A schematic representation of the genomic restriction map of the RET proto-oncogene and the probes that we have used is shown in Figure 1.

We have analysed 458 neoplastic samples, 40 thyroid and 418 non-thyroid, for RET activation by probing Southern blots with a 1 Kbp BglII-*Bam*H1 RET specific DNA fragment. This fragment is able to detect the region within the RET gene where the rearrangement can occur (Probe 1 of Figure 1). This probe detects a 6.3 Kbp fragment after restriction with *Eco*R1, a 3.7 Kbp *Bam*H1 and a 9.3 Kbp

**Figure 1** Schematic representation of the genomic restriction map of the RET proto-oncogene. The approximate positions of the coding sequences for the transmembrane (TM) and tyrosine kinase (TK) domains are shown. Below the map are illustrated the DNA probes used in this study. The restriction sites shown are E: *Eco*R1; B: *Bam*H1; Bg: BglII.

*Hind*III fragments in normal human DNA (Santoro *et al.*, 1992). None of the 418 non-thyroid neoplastic tissues (oesophageal, stomach, colon, liver, lung, kidney, ovarian, breast and prostate carcinomas; fibro and osteosarcomas, leukaemia and lymphomas, pituitary and parathyroid adenomas, neuroblastomas, gliomas, pheocromocytomas, and insulinomas) showed any rearrangement of the RET oncogene (Table II). None of the ten non-papillary thyroid carcinomas scored positive. Conversely we have found that three out of 30 thyroid papillary carcinomas, collected in France, showed additional rearranged bands and this result was demonstrable with at least three different restriction enzymes (Figure 2, lanes 1, 7, 8, 9, 10, 11, and 12).

In order to further characterise this rearrangement we have also analysed these positive samples with a NH2-terminal proto-RET specific sequence (1.8 kbp *Bam*H1 DNA fragment; probe 2 of Figure 1). Two out of these three positive samples showed rearranged bands also when probed with probe 2 (data not shown). This result indicated that the RET

**Table II** Tumours scored negative for PTC activation by Southern blot analysis

| *Tumour* | *Genomic DNA* | *RT-PCR* |
|---|---|---|
| Lung carcinoma[a] | 35 | 22 |
| Gastric carcinoma | 45 | 23 |
| Breast carcinoma | 40 | 13 |
| Colon carcinoma | 37 | 2 |
| Ovarian carcinoma | 12 | – |
| Uterine carcinoma | 10 | 13 |
| Renal carcinoma | 10 | 10 |
| Hepatocellular carcinoma | 3 | 16 |
| Esophageal carcinoma | 45 | – |
| Gall bladder carcinoma | – | 1 |
| Choledocal carcinoma | – | 1 |
| Prostate carcinoma | – | 5 |
| Pancreatic carcinoma | – | 2 |
| Pituitary adenoma | 25 | – |
| Insulinoma | 3 | – |
| Parathyroid adenoma | 3 | – |
| Acute leukaemia | 25 | – |
| Chronic leukaemia | 25 | – |
| Non Hodgkin lymphoma | 44 | 2 |
| Glioma | 20 | – |
| Pheochromocytoma | 10 | – |
| Neuroblastoma | 15 | – |
| Other sarcomas | 21 | – |
| Total | 418 | 110 |

[a]25 of these lung carcinomas were small cell lung cancers.

**Table I** Primers for PCR amplification and probes used for Southern blotting

| | *RET/PTC* | *c-raf-1* |
|---|---|---|
| Forward primer | 5′-ACTGAAGTGCAAGGCACT-3′ | 5′-GATTTCCTGGATCATGTT-3′ |
| Reverse primer | 5′-AAGTTCTTCCGAGGGAATTC-3′ | 5′-GCTGGCACGGGGGTTTTC-3′ |
| Probes for Southern | 5′-CCAGCGTGACCATCGAG GATCCAAAGTGGGA-3′ | 5′-CTGATTCGCTGTGACTTCGAA TTGCAT-3′ |

**Figure 2** Activation of the RET oncogene in papillary thyroid carcinomas. Southern blot analysis of DNA extracted from three different neoplastic specimens (Panels **a**, **b**, and **c** respectively). Ten µg of DNA were digested with *Eco*R1, *Bam*H1 or *Hind*III restriction enzymes (Amersham Corp.), transferred to Nylon filters (Hybond N, Amersham Corp.) and hybridised to a 1.0 Kbp. *Bam*H1-BglII RET specific DNA probe (showed in Figure 1). (**a**) Lane 1: DNA from papillary carcinoma number 1 digested with *Eco*R1; Lanes 2 and 3: normal human DNA digested with *Eco*R1. (**b**) Lanes 4, 5 and 6: normal human DNA digested with *Eco*R1, *Bam*H1 and *Hind*III respectively; Lanes 7, 8 and 9: DNA from the papillary carcinoma number 2 digested respectively with *Eco*R1, *Bam*H1 and *Hind*III. (**c**) Lanes 10, 11 and 12: DNA from the papillary carcinoma number 3 digested with *Eco*R1, *Hind*III and *Bam*H1 respectively. The arrows indicate the size of the normal fragments.

sequence located upstream of the breakpoint was not deleted in these two cases. Since both the RET proto-oncogene and H4 map to the long arm of chromosome 10, we hypothesise the possibility that a chromosomal inversion could lead to the H4/RET fusion. In fact we have reported that an inversion (10) (q11.2-21.1) caused the H4/RET fusion in at least four cases of papillary thyroid carcinoma (Pierotti *et al.*, 1992). Moreover the three positive thyroid samples were analysed by RT-PCR as described before (Santoro *et al.*, 1992). Two of them showed amplification of a fragment of the expected size of 363 bp, confirming that in these cases the activation of RET was due to an H4/RET fusion (data not shown). In the other positive thyroid papillary carcinoma the activation of RET, demonstrated by Southern analysis, was probably due to its fusion to a gene different from H4.

### *Analysis of the RET/PTC transcripts*

To study the activation of the RET proto-oncogene in human tumours, we have also used the more sensitive RT-PCR-Southern blotting technique to analyse 110 non-thyroid human tumours. The results was that all tumours listed in Table II, carcinomas of lung, stomach, breast, colon, uterus, kidney, liver, pancreas, prostate, choledochal and gallbladder, and lymphomas, were negative for the RET/PTC transcript. Representative results are shown in Figure 3. RNA extracted from the RET/PTC-positive TPC-1 cell line was used as a positive control. From TPC-1 RNA a fragment of about 100 bp in length was amplified which hybridised to a PTC chimeric point detecting probe (Figure 3a and b, lane 15) whereas in Figure 3 we show that 14 samples of breast carcinomas and 14 hepatocellular carcinomas gave no signal for the RET/PTC trancript (Figure 3a and b, lanes 1-14). To exclude the possibility that cDNAs were not amplified because of RNA degradation in these samples, c-*raf*-1 cDNA was amplified in the same reaction tube in which the RET/PTC cDNA was amplified and probed to a c-*raf*-1 specific oligonucleotide. A cDNA fragment of the expected size (about 500 bp) was amplified from all samples except for two samples of breast carcinoma (Figure 3a, lanes 8 and 9). These two samples were omitted for evaluating the involvement of RET/PTC.

### Discussion

The RET transforming gene has been found activated *in vivo* only in papillary thyroid carcinoma (Fusco *et al.*, 1987*a*; Grieco *et al.*,1990; Santoro *et al.*, 1992; Jhiang *et al.*,1992; Wajjwalku *et al.*, 1992), in a papillary thyroid carcinoma cell line (Ishizaka *et al.*, 1990), in four follicular thyroid adenomas and in one adenomatous goiter (Ishizaka *et al.*, 1991). No RET activation has been described in non-thyroid tumours apart from some cases in which RET rearrangements occurred during the transfection procedure (Takahashi *et al.*, 1985; Koda, 1988; Ishizaka *et al.*, 1989).

In this paper we confirm the frequency of about 10% of RET rearrangement in thyroid tumours from France, as previously described (Santoro *et al.*, 1992) and we report that no RET activation can be detected in 528 neoplasias of non-thyroid origin either by Southern blot analysis or by the much more sensitive PCR technique. However it is noteworthy that with an average frequency of RET-activation of 10%, the probability that no positive case will be found in the tumour groups smaller than 30 samples, just through random sampling error, is larger than 0.05. Some tumours have been examined with a too limited number of samples (<30) in this study therefore to draw statistically significant conclusions. Moreover of course, we cannot exclude the possibility that RET is activated in non-thyroid neoplasias that have not been analysed at all in this study. It is also possible that mechanisms other than the gene rearrangement described in thyroid tumours, for example point mutations, could activate RET in non-thyroid neoplasms, but it is worthwhile to mention that all the activated versions of RET described to date which occurred either *in vivo* or *in vitro* were due to gene rearrangement (see above). Finally a limitation of the RT-PCR assay, employed in this study, is that it

**Figure 3** Representative results of RT-PCR followed by Southern blotting. The primers shown in Table I were used for amplifying a fragment of the RET/PTC cDNA. For evaluating the quality of each RNA a part of c-*raf*-1 gene was amplified. TPC-1 (lane 15) was used as a positive control. (**a**) RT-PCR on breast carcinomas. (**b**) RT-PCR on hepatocellular carcinomas.

is able to detect only the fusion of RET to H4 and recently cases in which the activation of RET, in thyroid tumours, were caused by fusion to genes different from H4 have been reported, however these cases seem to represent less than 30% of all the RET-positive cases (Bongarzone *et al.*, 1993).

In conclusion these results suggest that RET activation is a molecular event linked only to thyroid neoplasias. Two hypotheses can be envisaged to explain this finding: the RET activation may occur only in thyroid cells, or this event might also occur in other cells, but it is unable to drive cells of non-thyroid origin to the neoplastic phenotype. The generation of transgenic mice carrying an activated RET oncogene, under the transcriptional control of the metallothioneine-promoter or the MMTV long terminal repeat, demonstrated induction of melanocytic tumours, mammary gland adenocarcinomas and other non-thyroid tumours (Iwamoto *et al.*, 1990; Iwamoto *et al.*, 1991). Thus the first hypothesis seems more likely. The restriction of the activation of RET to the thyroid suggests that this oncogene could act on a specific pathway in thyroid cells. Thus it will be extremely useful to study the biological activity of the RET/PTC products in two established differentiated rat thyroid cell lines that are available in our laboratory (Ambesi-Impiombato *et al.*, 1980; Fusco *et al.*, 1987*b*). Recently we have demonstrated that the introduction of RET/PTC is able to block the expression of the thyroid differentiated functions in the PC CL 3 rat thyroid cell line (Santoro *et al.*, 1993). We hope that the analysis of this cell line will be helpful to elucidate the pathway of action of the RET oncogene into the thyroid cell system.

We are grateful to Drs Shunzou Kobayashi (Nagoya City University), Hiroyuki Tsuda (Nagoya City University), Ryuichi Yatani (Mie University) and Yoshinori Murakami (National Cancer Center Research Institute) for providing us with tumour samples of breast carcinomas, renal cell carcinomas, prostate carcinomas and hepatocellular carcinomas. We also thank the Pathology Division of the National Cancer Center, (S. Sachie) for helping to collect tumour samples. We thank Dr Boiocchi for providing us with DNAs extracted from leukaemia and lymphomas. Dr Anglard for providing us with renal carcinoma samples, and Dr Berger and Dr Peix for providing us with thyroid tumours. Finally we thank Dr Umberto Giani for his comments about the statistical interpretation of the data.

This study was supported by the Associazione Italiana per la Ricerca sul Cancro (AIRC), the Progetto Finalizzato Ingegneria Genetica del CNR, the Progetto Finalizzato CNR ACRO-Sotto-progetto 2, Biologia Molecolare, a Grant-in-Aid from Cancer Research from the Ministry of Education, Science and Culture of Japan, and by La Ligue du Rhone contre le Cancer, Dr Sabino was recipient of an AIRC fellowship.

### References

AMBESI-IMPIOMBATO, F.S., PARKS, I.A.M. & COON, H.G. (1980). Culture of hormone-dependent functional epithelial cells from rat thyroids. *Proc. Natl Acad. Sci. USA*, **77**, 3455-3459.

BONGARZONE, I., PIEROTTI,M.A., MONZINI, N., MONDELLINI, P., MANENTI, G., DONGHI, R., PILOTTI, S., GRIECO, M., SANTORO, M., FUSCO, A., VECCHIO, G. & DELLA PORTA, G. (1989). High frequency of oncogene activation in human thyroid papillary carcinomas. *Oncogene*, **4**, 1457-1462.

BONGARZONE, I., MONZINI, N., BORRELLO, M.G., CARCANO, C., FERRARESI, G., ARIGHI, E., MONDELLINI, P., DELLA PORTA, G. & PIEROTTI, M.A. (1993). Molecular characterization of a thyroid tumor-specific transforming sequence formed by the fusion of ret tyrosine-kinase and the regulatory subunit RIalpha of cyclic AMP protein kinase A. *Mol. Cell. Biol.*, **13**, 358-366.

BONNER, T.I., OPPERMANN, H., SEEBURG, P., KERBY, S.B., GUNNELL, M.A., YOUNG, A.C. & RAPP, U.R. (1986). The complete coding sequence of the human raf oncogene and the corresponding structure of the c-*raf*-1 gene. *Nucleic Acids Res.*, **14**, 1009-1015.

CHOMCZIYNSKI, P. & SACCHI, N. (1987). Single step method of RNA isolation by acid guanidinium thiocyanate-phenol-chloroform extraction. *Anal. Biochem.*, **162**, 156-159.

FUSCO, A., GRIECO, M., SANTORO, M., BERLINGIERI, M.T., PILOTTI, S., PIEROTTI, M.A., DELLA PORTA, G. & VECCHIO, G. (1987*a*). A new oncogene in human papillary thyzoiol carcinomas and their lymph-nodal metastases. *Nature*, **328**, 7072.

FUSCO, A., BERLINGIERI, M.T., DI FIORE, P.P., PORTELLA, G., GRIECO,M. & VECCHIO, G. (1987*b*). One and two-step transformation of rat thyroid epithelial cells by retroviral oncogenes. *Mol. Cell. Biol.*, **7**, 3365-3370.

GRIECO, M., SANTORO, M., BERLINGIERI, M.T., MELILLO, R.M., DONGHI, R., BONGARZONE, I., PIEROTTI, M.A., DELLA PORTA, G., FUSCO, A. & VECCHIO, G. (1990). PTC is a novel rearranged form of the *ret* proto-oncogene and is frequently detected *in vivo* in human thyroid papillary carcinomas. *Cell*, **60**, 557-563.

ISHIZAKA, Y., OCHIAI, M., TAHIRA, T., SUGIMURA, T. & NAGAO, M. (1989). Activation of the *retII* oncogene without a sequence encoding a transmembrane domain and tranforming activity of two *retII* oncogene products differing in carboxytermini due to alternative splicing. *Oncogene*, **4**, 789–794.

ISHIZAKA, Y., USHIJIMA, T., SUGIMURA, T. & NAGAO, M. (1990). cDNA cloning and characterization of *ret* activated in a human papillary thyroid carcinoma cell line. *Biochem. Biophys. Res. Commun.*, **68**, 402–408.

ISHIZAKA, Y., KOBAYASHI, S., USHIJIMA, T., HIROHASHI, S., SUGIMURA, T. & NAGAO, M. (1991). Detection of retTPC/PTC transcripts in thyroid adenomas and adenomatous goiter by an RT-PCR method. *Oncogene*, **6**, 667–672.

ISHIZAKA, Y., SHIMA, H., SUGIMURA, T. & NAGAO, M. (1992). Detection of phosphorilated RET/PTC oncogene product in cytoplasm. *Oncogene*, **7**, 1441–1444.

IWAMOTO, T., TAKAHASHI, M., ITO, M., HAMAGUCHI, M., ISOBE, K., MISAWA, N., ASAI, J., YOSHIDA, T. & NAKASHIMA, I. (1990). Oncogenicity of the *ret* transforming gene in MMTV/ret transgenic mice. *Oncogene*, **5**, 533–542.

IWAMOTO, T., TAKAHASHI, M., ITO, M., HAMATANI, K., OHBAYASHI, M., WAJJWALKU, W., ISOBE, K. & NAKASHIMA, I. (1991). Aberrant melanogenesis and melanocytic tumour development in transgenic mice that carry a metallothionein/*ret* fusion gene. *EMBO J.*, **10**, 3167–3175.

JHIANG, S.M., CARUSO, D.R., GILMORE, E., ISHIZAKA, Y., TAHIRA, T., NAGAO, M. & MAZZAFERRI, E.L. (1992). Detection of the PTC/ret oncogene in human thyroid cancers. *Oncogene*, **7**, 1331–1337.

KAWASAKI, E.S. (1990). A guide to methods and applications. In *PCR Protocols Amplification of RNA*. Innis, M.A., Gelfand, D.H., Sninnski, J.J. & White, T.J. (eds), Academic Press, Inc.: San Diego, 21–38.

KODA, T. (1988). Ret gene from a human stomach cancer. *Hokkaido J. Med. Sci.*, **63**, 913–924.

LANZI, C., BORRELLO, M.G., BONGARZONE, I., MIGLIAZZA, A., FUSCO, A., GRIECO, M., SANTORO, M., GAMBETTA, R.A., ZUNINO, F., DELLA PORTA, G. & PIEROTTI, M.A. (1992). Identification of the product of two oncogenic rearranged forms of the RET proto-oncogene in papillary thyroid carcinomas. *Oncogene*, **7**, 2189–2194.

PIEROTTI, M.A., SANTORO, M., JENKINS, R.B., SOZZI, G., BONGARZONE, I., GRIECO, M., MONZINI, N., MIOZZO, M., HERRMANN, M.A., FUSCO, A., HAY, I.D., DELLA PORTA, G. & VECCHIO, G. (1992). Characterization of a chromosome 10q inversion juxtaposing RET and H4 genes and creating the oncogenic sequence PTC. *Proc. Natl Acad. Sci. USA*, **89**, 1616–1620.

SAMBROOK, J., FRITSCH, E.F. & MANIATIS, T. (1989). *Molecular Cloning: A Laboratory Manual*, Cold Spring Harbor, New York: Cold Spring Harbor Laboratory.

SANTORO, M., CARLOMAGNO, F., HAY, I.D., HERRMANN, M.A., GRIECO, M., MELILLO, R., BONGARZONE, I., PIEROTTI, M.A., DELLA PORTA, G., BERGER, N., PEIX, J.L., PAULIN, C., FABIEN, N., VECCHIO, G., JENKINS, R.B. & FUSCO, A. (1992). RET oncogene activation in human thyroid neoplasms is restricted to the papillary carcinoma subtype. *J. Clinical Investigation*, **89**, 1517–1522.

SANTORO, M., MELILLO, R.M., GRIECO, M., BERLINGIERI, M.T., VECCHIO, G. & FUSCO, A. (1993). The *TRK* and *RET* tyrosine kinase oncogenes cooperate with RAS in the neoplastic transformation of a rat thyroid epithelial cell line. *Cell Growth Diff.* (in press).

TAHIRA, T., ISHIZAKA, Y., ITOH, F., SUGIMURA, T. & NAGAO, M. (1990). Characterization of *ret* proto-oncogene mRNA encoding two isoforms of the protein product in a human neuroblastoma cell line. *Oncogene*, **5**, 97–102.

TAKAHASHI, M., RITZ, J. & COOPER, G.M. (1985). Activation of a novel human transforming gene, *ret*, by DNA rearrangement. *Cell*, **42**, 581–588.

TAKAHASHI, M. & COOPER, G.M. (1987). Ret transforming gene encodes a fusion protein homologous to tyrosine kinases. *Mol. Cell. Biol.*, **7**, 378–385.

TAKAHASHI, M., BUMA, Y., IWAMOTO, T., INAGUMA, Y., IKEDA, H. & HIAI, H. (1988). Cloning and expression of the *ret* proto-oncogene encoding a tyrosine kinase with two potential transmembrane domains. *Oncogene*, **3**, 571–578.

TAKAHASHI, M., BUMA, Y. & TANIGUCHI, M. (1991). Identification of the ret proto-oncogene products in neuroblastoma and leukemia cells. *Oncogene*, **6**, 297–301.

WAJJWALKU, W., NAKAMURA, S., HASEGAWA, Y., MIYAZAKI, K., SATOH, Y., FUNAHASHI, H., MATSUYAMA, M. & TAKAHASHI, M. (1992). Low frequency of rearrangement of the *ret* and *trk* proto-oncogenes in Japanese thyroid papillary carcinomas. *Jpn. J. Cancer Res.*, **83**, 671–675.